# Non-cryopreserved Autologous Peripheral Blood Stem Cell Transplantation for Lymphoma in a Resource-Limited Setting: Feasibility, Engraftment Kinetics, and Early Outcomes From Kashmir, India

**DOI:** 10.7759/cureus.110324

**Published:** 2026-06-05

**Authors:** Mohmad Hussain Mir, Suhail Ahmad Wani, Sheikh Muzamil Manzoor, Ajas Ibrahim, Farhana Siraj, Nisar Ahmad Syed, Faisal R Guru, Zeeza H Shah, Jaskaran Singh Sarao, Abrar Rasool, Afshan Atta, Malik T Rasool, Mushtaq Ahmad Sofi, Rubiya Ryhan, Mohd Zubair Qureshi, Sumyra Khurshid Qadri, Sunil K Regmi, Ulfat Ara, Shashwat Lohia, Aamir Sultan Lone, Nazir Ahmad Dar

**Affiliations:** 1 Department of Medical Oncology, Sher-i-Kashmir Institute of Medical Sciences, Srinagar, IND; 2 Department of Internal Medicine, Sher-i-Kashmir Institute of Medical Sciences, Srinagar, IND; 3 Department of Hematopathology, Sher-i-Kashmir Institute of Medical Sciences, Srinagar, IND; 4 Department of Radiation Oncology, Sher-i-Kashmir Institute of Medical Sciences, Srinagar, IND; 5 Department of Blood Transfusion and Immunohematology, Sher-i-Kashmir Institute of Medical Sciences, Srinagar, IND; 6 Department of Pathology, Sher-i-Kashmir Institute of Medical Sciences, Srinagar, IND

**Keywords:** autologious stem cell transplant, engraftment, kashmir, lymphoma, modified beam, non-cryopreserved stem cells, peripheral blood stem cells, resource-limited setting

## Abstract

Background

Autologous stem cell transplantation (ASCT) is a standard consolidation strategy for selected patients with relapsed or high-risk lymphoma, but access remains limited in many low- and middle-income regions because of the cost and infrastructure required for stem cell cryopreservation. Non-cryopreserved ASCT using fresh peripheral blood stem cells stored at 4°C may provide a practical alternative.

Methods

We retrospectively analyzed 11 consecutive patients with lymphoma who underwent non-cryopreserved ASCT at the Department of Medical Oncology, Sher-i-Kashmir Institute of Medical Sciences (SKIMS), Srinagar. Peripheral blood stem cells were mobilized with granulocyte colony-stimulating factor (G-CSF) alone or G-CSF plus plerixafor, stored at 4°C, and infused after institutional modified BEAM conditioning. Primary endpoints were feasibility, CD34+ cell collection, neutrophil and platelet engraftment, early toxicities, and day +100 survival.

Results

The median age was 41 years (range, 22-58), and 9 patients (81.8%) were male. Diagnoses included classical Hodgkin lymphoma in 4 patients (36.4%) and non-Hodgkin lymphoma in 7 patients (63.6%). Stage III-IV disease was present in 8 patients (72.7%), and 9 patients (81.8%) were in complete remission before transplant. G-CSF plus plerixafor was used in 9 patients (81.8%). The median CD34+ cell dose collected was 5.5 × 10^6/kg (range, 3.6-13.5), with a median of 2 apheresis sessions (range, 1-2). All grafts were stored for 6 days at 4°C. Median neutrophil and platelet engraftment occurred on day 11 (range, 10-13) and day 12 (range, 10-18), respectively. Febrile neutropenia occurred in 10 patients (90.9%); grade 3-4 mucositis occurred in 6 patients (54.5%). Day +30 survival was 100%, and day +100 survival was 90.9%, with one transplant-related death.

Conclusion

Non-cryopreserved ASCT for lymphoma was feasible at SKIMS Srinagar, with adequate CD34+ cell collection, rapid engraftment, and acceptable early survival. Larger prospective studies with standardized graft-quality monitoring, longer follow-up, and cost-effectiveness analysis are needed.

## Introduction

High-dose chemotherapy followed by autologous stem cell transplantation (ASCT) is an established therapeutic strategy for selected patients with chemosensitive relapsed or refractory Hodgkin lymphoma and aggressive non-Hodgkin lymphoma. Its benefit over conventional-dose salvage approaches was shown in pivotal lymphoma trials, and ASCT remains incorporated into contemporary transplant practice recommendations for appropriately selected patients [1,2,3]. Peripheral blood stem cells have largely replaced bone marrow as the preferred autologous graft source because they permit faster hematopoietic recovery after myeloablative chemotherapy [4].

The conventional ASCT platform requires mobilization and apheresis, graft processing, cryopreservation using dimethyl sulfoxide (DMSO), storage in liquid nitrogen, thawing, and reinfusion after conditioning chemotherapy. This approach is reliable and provides scheduling flexibility, but it requires controlled-rate freezing, liquid nitrogen storage, continuous temperature monitoring, trained staff, quality systems for sterility and viability, and safe handling of DMSO [5,6]. These requirements increase cost and complexity and may restrict access in low- and middle-income regions where transplant need is high but infrastructure is unevenly distributed.

Non-cryopreserved ASCT is a resource-adapted alternative in which harvested peripheral blood stem cells are stored at 4°C for a limited period and reinfused after completion of conditioning. A systematic review by Wannesson L et al. supported the feasibility and safety of autotransplants using non-cryopreserved marrow or peripheral blood stem cells, and subsequent disease-specific experiences extended this approach to multiple myeloma and lymphoma [7,8]. Early reports from Egypt, Colombia, India, and other resource-constrained settings showed reliable engraftment using fresh autologous grafts, with potential reductions in cost, processing complexity, and DMSO-related toxicity [9,10,11].

The evidence base has expanded through comparative and observational studies reporting acceptable engraftment and early outcomes with non-cryopreserved grafts in myeloma and lymphoma, including experiences using BEAM-like regimens and adapted lymphoma conditioning schedules [12,13,14]. The Worldwide Network for Blood and Marrow Transplantation (WBMT) has summarized practice considerations for non-cryopreserved autologous peripheral blood stem cell transplantation in countries with limited resources, emphasizing careful logistics, adequate CD34+ collection, short and controlled storage, and strict quality oversight [15].

Lymphoma represents a more demanding setting for non-cryopreserved ASCT than myeloma because BEAM or BEAM-like conditioning usually extends over several days, requiring grafts to retain functional viability during refrigerated storage. Adequate mobilization is therefore central to this platform; consensus guidance supports risk-adapted use of agents such as plerixafor to improve collection success, particularly when timely reinfusion is required. Published data from North India, particularly Kashmir, remain scarce. We therefore analyzed the feasibility, engraftment kinetics, transplant-related toxicities, and early outcomes of non-cryopreserved ASCT for lymphoma performed at the Department of Medical Oncology, Sher-i-Kashmir Institute of Medical Sciences (SKIMS), Srinagar, India.

## Materials and methods

Study design and setting

This was a retrospective single-center analysis of patients with lymphoma who underwent non-cryopreserved ASCT at the Department of Medical Oncology, Sher-i-Kashmir Institute of Medical Sciences (SKIMS), Srinagar, India. The analysis used deidentified institutional data. Ethical clearance was sought for conducting this retrospective study under the broader application to evaluate the pattern, distribution, and features of hematolymphoid malignancies in Kashmir over the last decade: a 10-year retrospective single-center observational study at SKIMS from 2013 to 2023.

Patients

Eligible patients had lymphoma and underwent non-cryopreserved ASCT using peripheral blood stem cells. The cohort included patients with relapsed/refractory classical Hodgkin lymphoma and non-Hodgkin lymphoma. Baseline demographic data, lymphoma subtype, stage, prior treatment, pretransplant disease status, mobilization strategy, CD34+ cell collection, transplant-related variables, and early survival outcomes were abstracted from the institutional dataset. Response status was recorded from institutional documentation; where possible, disease response definitions were aligned with the Lugano criteria.

Stem cell mobilization, collection, and storage

Stem cells were mobilized using G-CSF alone or G-CSF plus plerixafor. No patient underwent chemomobilization in the analyzed cohort. Apheresis was performed until an adequate CD34+ cell dose was collected. Products were stored without cryopreservation at 4°C and reinfused following completion of conditioning. The available dataset did not include graft viability or sterility-culture results.

Conditioning and supportive care

All patients received an institutional modified BEAM conditioning regimen. The standard dosing schedule of drugs was used. G-CSF support was used after transplant. Antimicrobial therapy and transfusion support were provided according to institutional practice. Toxicities were summarized using the worst recorded mucositis grade, febrile neutropenia, duration of antibiotic therapy, and blood product support.

Definitions

Neutrophil engraftment was defined as the first day of neutrophil recovery documented in the transplant dataset. Platelet engraftment was defined as the first day of platelet recovery documented in the dataset. Early outcomes were assessed at day +30 and day +100. Transplant-related mortality (TRM) was defined as death by day +100 that was not clearly attributable to lymphoma progression.

Statistical analysis

Continuous variables were summarized as medians with ranges. Categorical variables were summarized as frequencies and percentages. Because of the small sample size, no formal hypothesis testing was performed. The study was designed as a feasibility analysis rather than a comparative efficacy study.

## Results

Baseline characteristics

Eleven patients were included. The median age was 41 years (range, 22-58); 9 patients (81.8%) were male and 2 (18.2%) were female. Classical Hodgkin lymphoma accounted for 4 cases (36.4%), and non-Hodgkin lymphoma accounted for 7 (63.6%). Non-Hodgkin lymphoma subtypes included diffuse large B-cell lymphoma, peripheral T-cell lymphoma not otherwise specified, ALK-negative anaplastic large-cell lymphoma, and blastoid-variant mantle cell lymphoma. Stage III-IV disease was present in 8 patients (72.7%). Before ASCT, 9 patients (81.8%) were in complete remission and 2 (18.2%) were in partial response (Table 1).

**Table 1:** Baseline characteristics of the cohort. ASCT: Autologous stem cell transplantation.

Characteristic	Value
Total patients	11
Age, years, median (range)	41 (22-58)
Male/Female	9 (81.8%) / 2 (18.2%)
Classical Hodgkin lymphoma	4 (36.4%)
Non-Hodgkin lymphoma	7 (63.6%)
Stage III-IV disease	8 (72.7%)
Complete remission before ASCT	9 (81.8%)
Partial response before ASCT	2 (18.2%)

Mobilization and graft characteristics

G-CSF plus plerixafor was used in 9 patients (81.8%), while G-CSF alone was used in 2 (18.2%). No patient received chemomobilization. The median number of apheresis sessions was 2 (range, 1-2). The median collected CD34+ cell dose was 5.5 × 10^6^/kg (range, 3.6-13.5). All grafts were stored at 4°C for 6 days before infusion.

Engraftment

Hematopoietic recovery was rapid. Median neutrophil engraftment occurred on day 11 (range, 10-13), and median platelet engraftment occurred on day 12 (range, 10-18). No primary graft failure was recorded in the dataset (Table 2).

**Table 2:** Mobilization, graft characteristics, engraftment, and early toxicity. G-CSF: Granulocyte colony-stimulating factor; TRM: Transplant-related mortality.

Parameter	Value
G-CSF + plerixafor mobilization	9 (81.8%)
G-CSF alone mobilization	2 (18.2%)
Chemomobilization	0 (0.0%)
Apheresis sessions, median (range)	2 (1-2)
Collected CD34+ cell dose, ×10^6^/kg, median (range)	5.5 (3.6-13.5)
Storage at 4°C, days	6 in all patients
Neutrophil engraftment, day, median (range)	11 (10-13)
Platelet engraftment, day, median (range)	12 (10-18)
Febrile neutropenia	10 (90.9%)
Antibiotic duration, days, median (range)	10 (1-15)
Grade 3-4 mucositis	6 (54.5%)
Day +30 survival	11 (100.0%)
Day +100 survival	10 (90.9%)
Day +100 TRM	1 (9.1%)

Toxicity and supportive care

Febrile neutropenia occurred in 10 patients (90.9%). The median antibiotic duration was 10 days (range, 1-15). The median packed red-cell requirement was 2 units (range, 0-5), the median single-donor apheresis platelet requirement was 4 units (range, 0-6), and the median random-donor platelet requirement was 6 units (range, 0-10). Mucositis was common; grade 3-4 mucositis was documented in 6 patients (54.5%).

Early survival

All patients were alive at day +30. At day +100, 10 patients (90.9%) were alive and one patient had died, giving a day +100 overall survival of 90.9% and a day +100 TRM of 9.1%. The patient who died had ALK-negative anaplastic large-cell lymphoma, stage IV disease, complete response before transplant, neutrophil engraftment on day 13, platelet engraftment on day 17, and grade 4 mucositis. The immediate cause of death was infection with bilateral lung consolidations and type 1 respiratory failure, and the patient succumbed to these complications. The detailed deidentified transplant summary is tabulated below (Table 3).

**Table 3:** Deidentified patient-level transplant summary. cHL: Classical Hodgkin lymphoma; ALK: Anaplastic lymphoma kinase; ALCL: Anaplastic large-cell lymphoma; DLBCL non-GCB: Diffuse large B-cell lymphoma, non-germinal center B-cell type; PTCL-NOS: Peripheral T-cell lymphoma, not otherwise specified; MCL blastoid: Blastoid-variant mantle cell lymphoma; CR: Complete remission or complete response; PR: Partial remission or partial response; ANC: Absolute neutrophil count; Plt: Platelets.

No.	Age/sex	Dx	Stage	Pre-ASCT	CD34 ×10^6^/kg	Harvests	ANC/Plt engraftment day	Worst mucositis grade	Day +100
1	22/F	cHL	III, bulky mediastinal	CR	5.6	2	13/17	3	Alive
2	25/M	cHL	II, bulky unfavorable	PR	4.8	2	11/16	2	Alive
3	49/M	cHL	III	CR	5.9	2	12/18	3	Alive
4	30/M	ALK-negative ALCL	IV	CR	4.5	2	13/17	4	Died
5	50/F	PTCL-NOS	III	CR	3.8	1	12/14	4	Alive
6	39/M	cHL	IV	CR	3.6	1	10/12	3	Alive
7	41/M	DLBCL, non-GCB	II, bulky	CR	6.1	2	10/11	2	Alive
8	58/M	PTCL-NOS	IV	CR	13.5	1	10/10	3	Alive
9	35/M	DLBCL, non-GCB	II, bulky	PR	5.5	2	10/10	2	Alive
10	50/M	DLBCL, GCB	III	CR	3.72	2	11/12	1	Alive
11	57/M	Blastoid MCL	IV	CR	8.2	1	10/11	1	Alive

## Discussion

This single-center experience shows that non-cryopreserved ASCT for lymphoma is feasible at SKIMS Srinagar using fresh peripheral blood stem cells stored at 4°C. The platform achieved a median CD34+ cell dose of 5.5 × 10^6^/kg, no primary graft failure, median neutrophil recovery by day 11, and median platelet recovery by day 12. These engraftment kinetics are within the range reported in earlier non-cryopreserved ASCT series and are clinically comparable to expected recovery after standard cryopreserved autologous transplantation [7,9-11,14-19].

The principal rationale for a non-cryopreserved platform is pragmatic rather than experimental. Cryopreservation is effective, but it requires infrastructure, quality control, trained personnel, liquid nitrogen, DMSO exposure, and reliable power and monitoring systems [5,6]. These requirements increase cost and restrict scalability in regions where patients with lymphoma may otherwise be eligible for curative-intent or disease-consolidating ASCT. Several resource-limited programs have reported that avoiding cryopreservation can simplify workflow and reduce cost without compromising early hematopoietic recovery [9,10,11,14-17,19].

Our results are particularly relevant because lymphoma conditioning is longer and more complex than the single-agent melphalan schedules used in myeloma. Earlier lymphoma experiences from Egypt and Colombia demonstrated that non-cryopreserved grafts can support hematopoiesis after multiagent conditioning, including BEAM- or CBV-based approaches [9,10,11,16]. Larger and more recent cohorts from India, Thailand, Algeria, and Chile have further supported the feasibility of refrigerated graft storage and reinfusion after lymphoma conditioning [14,15,17,19]. The present SKIMS cohort adds data from Kashmir, a landlocked Himalayan region in North India, and supports the applicability of this approach in a North Indian public-sector tertiary care setting with very limited resources for cryopreservation.

The median collected CD34+ cell dose in our cohort was adequate and likely contributed to rapid engraftment. Most patients received G-CSF plus plerixafor, which may have reduced the risk of collection failure and allowed the transplant sequence to proceed without delay. Although plerixafor increases upfront mobilization cost, consensus guidance supports risk-adapted use when collection failure is anticipated or when timely collection is essential [20]. In a non-cryopreserved platform, successful first-attempt mobilization may prevent failed apheresis, conditioning delays, or cancellation after the transplant pathway has begun; future cost analyses should therefore evaluate total episode-of-care costs rather than mobilization cost alone.

Toxicity deserves balanced interpretation. Febrile neutropenia occurred in 90.9% of patients, and grade 3-4 mucositis occurred in more than half of the cohort. These findings are consistent with the expected toxicity of BEAM-like conditioning, but they indicate that non-cryopreservation does not remove the need for high-quality supportive care, antimicrobial stewardship, transfusion support, and early sepsis management. One day +100 transplant-related death occurred, giving a TRM of 9.1%. Because the denominator is small, this estimate is unstable; however, it is clinically important and was reported transparently, with the cause of death being pneumonia and type 1 respiratory failure due to sepsis.

The absence of primary graft failure in this cohort is reassuring. Published non-cryopreserved studies have reported generally low graft failure rates when adequate CD34+ doses are infused and storage is controlled [11,13,17,19,21]. Nevertheless, graft viability testing, sterility cultures, and temperature monitoring are essential quality components, especially when storage extends across several days for lymphoma conditioning. These variables should be incorporated into routine institutional documentation and future prospective studies.

The broader literature supports cautious adoption rather than unqualified equivalence. Comparative studies in myeloma and mixed-disease cohorts suggest similar engraftment between cryopreserved and non-cryopreserved grafts, with possible advantages in cost, hospitalization, and avoidance of DMSO-related toxicity [18,22]. However, the evidence is largely retrospective or observational, and the WBMT practice-considerations paper emphasizes the absence of definitive randomized data [15]. Therefore, our findings should be interpreted as feasibility evidence from a resource-limited center rather than proof that fresh grafts are universally equivalent to cryopreserved grafts.

This study has some limitations. The sample size is small, the design is retrospective, and there is no contemporaneous cryopreserved control group. We also could not analyze the impact of comorbidity using a formal transplant comorbidity index, which has prognostic value in transplant populations [23]. Despite these limitations, the SKIMS experience provides useful regional evidence. It demonstrates that, with careful patient selection, adequate CD34+ collection, coordinated conditioning, and reliable supportive care, non-cryopreserved ASCT can be delivered for lymphoma patients in a resource-limited setting. The approach may help expand access to transplantation in centers where cryopreservation facilities are unavailable or unaffordable, but prospective multicenter studies with standardized graft-quality monitoring, toxicity reporting, cost-effectiveness analysis, and longer follow-up are needed before wider policy-level recommendations are made [15,24,25].

## Conclusions

Non-cryopreserved autologous peripheral blood stem cell transplantation for lymphoma was feasible at SKIMS Srinagar, with adequate CD34+ collection, rapid neutrophil and platelet engraftment, and acceptable day +100 survival. This approach may provide a practical platform for expanding lymphoma ASCT in resource-limited settings. However, clinically significant mucositis and one transplant-related death emphasize the need for careful patient selection, meticulous supportive care, and prospective quality-control data. Larger multicenter studies with longer follow-up and cost-effectiveness analysis are warranted.
